# A 3-biomarker-panel predicts renal outcome in patients with proteinuric renal diseases

**DOI:** 10.1186/s12920-014-0075-8

**Published:** 2014-12-24

**Authors:** Hannes Neuwirt, Paul Perco, Alexander Kainz, Irmgard Mühlberger, Johannes Leierer, Suzie-Jane Braniff, Bernd Mayer, Gert Mayer, Michael Rudnicki

**Affiliations:** Department of Internal Medicine IV - Nephrology and Hypertension, Medical University Innsbruck, Anichstrasse 35, 6020 Innsbruck, Austria; Emergentec biodevelopment GmbH, Vienna, Austria; Department of Internal Medicine III – Nephrology, KH Elisabethinen, Linz, Austria

**Keywords:** Histogenomics, Transcriptomics, Microarray, Biomarker, Chronic kidney disease, Prognosis, Bioinformatics

## Abstract

**Background:**

Clinical and histological parameters are valid prognostic markers in renal disease, although they may show considerable interindividual variability and sometimes limited prognostic value. Novel molecular markers and pathways have the potential to increase the predictive prognostic value of the so called “traditional markers”.

**Methods:**

Transcriptomics profiles from laser-capture microdissected proximal tubular epithelial cells from routine kidney biopsies were correlated with a chronic renal damage index score (CREDI), an inflammation score (INSCO), and clinical parameters. We used data from 20 renal biopsies with various proteinuric renal diseases with a median follow-up of 49 months (discovery cohort). For validation we performed microarrays from whole kidney biopsies from a second cohort consisting of 16 patients with a median follow-up time of 28 months (validation cohort).

**Results:**

562 genes correlated with the CREDI score and 285 genes correlated with the INSCO panel, respectively. 39 CREDI and 90 INSCO genes also correlated with serum creatinine at follow-up. After hierarchical clustering we identified 5 genes from the CREDI panel, and 10 genes from the INSCO panel, respectively, which showed kidney specific gene expression. After exclusion of genes, which correlated to each other by > 50% we identified VEGF-C from the CREDI panel and BMP7, THBS1, and TRIB1 from the INSCO panel. Traditional markers for chronic kidney disease progression and inflammation score predicted 44% of the serum creatinine variation at follow-up. VEGF-C did not further enhance the predictive value, but BMP7, THBS1 and TRIB1 together predicted 94% of the serum creatinine at follow up (p < 0.0001). The model was validated in a second cohort of patients yielding also a significant prediction of follow up creatinine (48%, p = 0.0115).

**Conclusion:**

We identified and validated a panel of three genes in kidney biopsies which predicted serum creatinine at follow-up and therefore might serve as biomarkers for kidney disease progression.

**Electronic supplementary material:**

The online version of this article (doi:10.1186/s12920-014-0075-8) contains supplementary material, which is available to authorized users.

## Background

In biopsy-proven renal diseases several clinical and histopathological features have been established as markers indicative of progression [1-5]. Nonetheless, most of these traditional risk markers have limited accuracy and reliability, which may be improved by including further molecular markers. Microarray technology and integrative bioinformatics strategies resulted in the identification of novel molecular features, multi-gene expression patterns, and biological pathways being associated with renal disease progression. In native kidney disease, Reich and colleagues identified a panel of eleven genes expressed in kidney biopsies which were related to the degree of proteinuria and allowed to distinguish biopsies from patients with IgA nephritis (IgAN) from control subjects [6]. Boettinger and co-workers identified a panel of 30 TGF-beta 1-related transcripts expressed in the renal tubulointerstitial compartment which correlated significantly with eGFR in patients with CKD I-V [7]. Our group showed that diminished renal tubular expression of VEGF-A and increased expression of hypoxia response genes at time of biopsy better predict renal outcome in CKD patients than serum creatinine and proteinuria [8]. In zero-hour renal allograft biopsies Perco et al. reported a panel of three genes enhancing the predictive value for allograft function one year after kidney transplantation by 2-fold as compared to traditional markers of allograft function [9]. Einecke et al. published a panel of 30 genes from “for cause”-kidney transplant biopsies which predicted graft loss better than pathohistological features or function at time of biopsy [10]. More recently, Sellares et al. developed a molecular score for antibody-mediated rejection in kidney grafts consisting of the expression values of 30 specific genes, which significantly predicted future graft failure [11]. Pathways that have been identified as highly affected in high-throughput Omics studies in the context of progressive native kidney disease included for example the *VEGF-signaling* and *hypoxia response* pathways in various glomerulonephritis [8], or the *NF-κB module NFKB_IRFF_01* pathway in diabetic nephropathy [12]. These studies further underline a substantial benefit of multi-gene over single-gene patterns. However, most gene expression studies on the association of renal transcriptomics and kidney function decline are cross-sectional with only a limited number of longitudinal studies available.

The condition of the tubulointerstitial compartment and in particular of proximal tubular epithelial cells (PTECs) plays a pivotal role in the progression of chronic renal failure. A variety of mechanisms possibly responsible for renal function decline have been identified in this context, such as tubular atrophy, intersititial fibrosis, tubulointerstitial hypoxia, capillary rarefaction, impaired angiogenesis, epithelial-mesenchymal transition and inflammation [13]. Importantly, these histopathological features can be found in various renal diseases, such as FSGS, minimal change disease, lupus nephritis or IgA nephropathy, independently from diagnosis and correlate with poor renal prognosis. Therefore, we and others focused on gene-expression profiles derived from microdissected PTECs or from the tubulointerstitial compartment [8,12].

In this project we followed a longitudinal study setup utilizing transcriptomics data from two of our recent studies [8,14]. The given gene expression sets derived from laser-capture micro dissected (LCM) human renal proximal tubule cells were correlated to histological characteristics and to renal function after a median follow up of 49 months to identify candidate markers for the prediction of renal disease progression. The results from the first cohort were validated in an independent cohort of whole kidney biopsies.

## Methods

### Renal biopsies, RNA isolation and microarray hybridization

In the current study we analyzed pre-existing microarray expression data of laser-capture microdissected (LCM) PTECs from 50 renal biopsy samples from patients with proteinuric kidney diseases (discovery cohort) from two of our previous studies [8,14]. Of these samples those with insufficient material for detailed histological assessment of renal damage and inflammation score (see below) were excluded. Additionally, we excluded those that were not on a stable dosing of immunosuppression, where applicable (e.g. Lupus nephritis), prior to biopsy or developed AKI within 1 week after biopsy. Finally, 27 renal biopsy samples (discovery cohort) fulfilled these criteria and were in depth analysed for histological signs of damage and inflammation (see below).

The degree of glomerular sclerosis (gs), interstitial fibrosis and tubular atrophy (ifta) as well as interstitial inflammation (ii) was assessed for each of the biopsies as follows: 0 = 0% (none), 1 = 1 – 10% (slight), 2 = 11 – 25%, 3 = 26 – 50% (moderate) and 4 > 50% (severe). The chronic renal damage index (CREDI) for each biopsy was derived from the sum score of gs and ifta. The degree of interstitial inflammation resulted directly in the inflammation score (INSCO). Follow-up laboratory data was available for 20 of these patients, with a median follow-up time of 49 months (range: 29 – 68 months).

A second group of patients with proteinuric kidney diseases was used for validation. We analyzed microarray expression data of whole kidney biopsies from 16 renal biopsy samples from patients with proteinuric kidney diseases (validation cohort). The degree of gs, ifta and ii was assessed for each of the biopsies as stated above. The median follow-up time was 28 months (range: 1 – 72 months).

In the discovery and the validation cohort progressive disease was defined as either doubling of serum creatinine or reaching end-stage renal disease during follow-up, all other patients were defined as stable. For data security purposes individual ages are not stated in the tables; instead age groups have been defined: group 1 = age <30 years, group 2 = age 30–45 years, group 3 = age 46–60 years, group 4 = age > 60 years. However, we used individual ages for statistics concerning age comparing progressive and stable patients.

The LCM, RNA isolation, and microarray hybridization have been described in detail in previous work [14,15]. In brief, PTECs were stained for alkaline phosphatase using 4-nitro blue tetrazolium chloride/5-bromo-4-chloro-3-indolyl phosphate under RNase-free conditions, and the cells were isolated using the PixCell IIs Laser Capture Microdissection System and CapSure™ LCM Caps (Arcturus, Mountain View, CA, USA). Total RNA was isolated using the Pico Pure™ RNA Isolation Kit (Arcturus, Mountain View, CA, USA). Owing to low RNA amounts, we performed two rounds of linear RNA amplification using the RiboAmp™ RNA Amplification Kit (Arcturus, Mountain View, CA, USA). The quality of the amplified RNA was assessed by spectrophotometry (A260/280) and with the Agilent Bioanalyzer and RNA6000 LabChip™ Kit (Agilent, Palo Alto, CA, USA). cDNA-microarrays were obtained from the Stanford Functional Genomics Facility (https://microarray.org/sfgf/). The arrays contained 41 792 spots, representing 30 325 genes assigned to a UniGene cluster and 11467 ESTs. Arrays were scanned using a GenePix 4000B microarray scanner, and the images were analyzed with the GenePix Pro 4.0 software (Axon Instruments, Union City, CA, USA). All samples were processed in technical duplicates and gene expression values were averaged.

The Institutional Review Board (IRB) of the Medical University of Innsbruck (Ethikkommittee Medizinische Universtität Innsbruck) accredited the use of surplus material from routine for-cause renal biopsies for research purposes. A written consent was not obtained (and not required from the IRB) as the biospies were performed in the context of clinical routine and have been analyzed after patient treatment and hospitalization were ultimately completed. All other patient records have been anonymized and de-identified prior to analysis.

### Establishment and validation of a biomarker model predictive for follow-up creatinine

#### Correlation of genome-wide gene expression with histological scores

We included genes with intensity values of more than 2.5 over background and a valid signal in more than 80% of the processed arrays in further analysis. Pearson correlation coefficients were calculated between gene expression values and the CREDI as well as the INSCO scores, respectively.

Significantly correlated genes (p-value < 0.01) with at least one of the two histological scores were functionally annotated using gene ontology terms as provided by the SOURCE tool (http://source.stanford.edu). We furthermore searched for biological pathways being enriched or depleted with respect to the set of significantly correlated genes using the PANTHER (Protein ANalysis THrough Evolutionary Relationships) Classification System [16-18].

#### Linear regression model for prediction of creatinine at follow-up

Univariate linear regression models including expression values of candidate genes were computed to predict serum creatinine values at follow-up. For genes showing a significant prediction (p < 0.05) kidney-specific gene expression was evaluated using information as provided by the SOURCE tool. We focused on kidney-specific genes by purpose as microarray data derived from laser-capture microdissected renal proximal tubule cells. From the set of candidate genes, the ones showing high correlation to serum creatinine but low Pearson correlation coefficients in their pairwise comparison (Pearson R < 0.5 and > −0.5, respectively) were selected for building multivariate regression models. This procedure was applied for avoiding the inclusion of highly correlated variables into one model. Multiple linear regression models for the prediction of kidney function were established using a combination of genes based on either the CREDI or the INSCO panel along with the gold standard parameters INSCO, CREDI, creatinine and proteinuria at time of biopsy. The best model was generated in a step-wise selection procedure. In order to get a 95% confidence interval for the coefficient of determination we applied a bootstrap sampling using 2000 case resampled datasets. The confidence interval was computed by the bias corrected algorithm [19].

For validation of the model found in the discovery cohort we performed linear regression analysis for the predictive value in a second cohort of patients (validation cohort).

For a better comparison of our data with published literature we also calculated sensitivity and specificity for each biomarker candidate using the renal endpoints “end stage renal disease (ESRD)” and “doubling of serum creatinine” for definition of progressive kidney disease (see Tables 1 and 2). Using Youden’s J statistics [20] (J = Sensitivity + Specificity – 1) we calculated the optimal cut-off values [micro array fluorescence intensity values] for each biomarkers with respect to maximized sensitivity and specificity.

**Table 1:** **Patient characteristics – discovery cohort**

**Patient**	**Age group**	**Follow-up time [months]**	**Creatinine at time of biopsy [mg/dl]**	**Creatinine at follow-up [mg/dl]**	**eGFR (MDRD) at time of biopsy [ml/min/1.73 m** ^**2**^ **]**	**eGFR (MDRD) at follow-up [ml/min/1.73 m** ^**2**^ **]**	**Proteinuria at time of biopsy [g/d]**	**Proteinuria at follow-up [g/d]**	**Histological diagnosis**	**SD/PD**	**Endpoint**	**IFTA**	**GS**	**IFTA + GS = CREDI**	**II = INSCO**
1	2	62	0.84	0.88	>60	>60	1.9	3.2	FSGS	Stable	-	2	2	4	0
2	2	49	0.76	0.84	>60	>60	4.7	4.7 mg/dl	MCD	Stable	-	0	0	0	0
3	4	63	0.79	0.75	>60	>60	500 mg/dl	0.0	MCD	Stable	-	1	1	2	1
4	2	43	1.34	1.09	58.1	>60	0.4	45.1 mg/dl	IGAN	Stable	-	1	2	3	1
5	1	49	0.77	0.76	>60	>60	7.4	43.0	FSGS	Stable	-	0	2	2	0
6	2	39	0.8	0.75	>60	>60	1.6	0.2	MCD	Stable	-	1	1	2	0
7	1	31	1.0	0.96	>60	>60	1.3	14.8 mg/dl	IGAN	Stable	-	1	1	2	1
8	3	52	0.93	0.97	>60	>60	1.3	263.3 mg/dl	IGAN	Stable	-	1	1	2	0
9	1	51	1.89	0.91	44.8	>60	42.0	42.0	FSGS	Stable	-	3	2	5	0
10	2	49	1.43	1.43	40.2	39.5	1.0	0.8	IGAN	Stable	-	3	3	6	1
11	2	50	1.56	1.8	51.8	42.9	1.2	NA	IGAN	Stable	-	2	2	4	0
12	3	67	1.42	2.28	51.6	29.3	2.7	4.6	FSGS	Stable	-	2	2	4	1
13	2	68	1.71	3.63	46.9	20.9	1.6	3.6	IGAN	Progressive	2xCrea	3	2	5	1
14	1	51	1.02	3.5	>60	21	12.0	17.5	FSGS	Progressive	2xCrea	2	3	5	2
15	1	29	1.20	1.81	54.3	33.3	7.5	3.2	IGAN	Stable	-	2	3	5	0
16	4	42	1.50	7.02	34.5	<20	8.5	8.0	MGN	Progressive	ESRD	2	2	4	1
17	1	49	1.87	10.1	33.4	<20	6.5	NA	RPGN	Progressive	ESRD	2	1	3	2
18	2	48	1.27	4.02	47.9	<20	6.5	1.3	LN4	Progressive	2xCrea	4	4	8	3
19	4	56	3.13	3.37	<20	<20	4.0	1.0	MPGN II	Stable	-	2	3	5	1
20	3	31	5.07	6.03	<20	<20	3.4	2.9	MPA	Progressive	ESRD	3	2	5	2
21	3	n.a.	4.67	NA	<20	NA	10.6	NA	IGAN	NA	NA	1	4	5	1
22	2	n.a.	0.71	NA	>60	NA	10.2	NA	FSGS	NA	NA	1	1	2	0
23	2	n.a.	1.12	NA	>60	NA	0.5	NA	IGAN	NA	NA	3	2	5	1
24	2	n.a.	1.09	NA	>60	NA	6.3	NA	FSGS	NA	NA	1	1	2	1
25	1	n.a.	1.0	NA	>60	NA	1.2	NA	IGAN	NA	NA	0	0	0	0
26	3	n.a.	1.4	NA	52.6	NA	10.0	NA	MCD	NA	NA	1	1	2	0
27	4	n.a.	2.44	NA	<20	NA	18.0	NA	MCD	NA	NA	2	1	3	1
Median	33	49	1.42	1.26			2.7	3.4				2.0	2.0	4.0	1.0
Mean	39	49	1.66	2.64			4.0	5.5				1.9	2.0	3.8	0.9
SD	17	11	1.12	2.65			2.9	6.0				1.0	0.9	1.8	0.9
Median S	33	50	1.38	0.96			1.9	3.9				1.5	2.0	3.5	0.0
Median P	33	49	1.71	6.03			6.5	3.3				2.5	2.0	5.0	2.0
Mean S	39	49	1.4	1.29			3.2	3.9				1.5	1.8	3.3	0.4
Mean P	40	48	2.19	6.15			5.8	6.3				2.7	2.3	5.0	1.8
SD S	17	11	0.72	0.78			2.7	1.0				NA	NA	NA	NA
SD P	19	12	1.65	2.59			2.9	7.5				NA	NA	NA	NA
p-value	n.s.	n.s.	n.s.	0.01			n.s.	n.s.				n.s.	n.s.	n.s.	0.004

FSGS Focal-segmental glomerulosclerosis, MCD Minimal change disease, IGAN IgA-Nephropathy, MGN Membranous glomerulonephritis, RPGN Rapid progressive glomerulonephritis, LN4 Lupus nephritis WHO IV, MPGN II Membranoproliferative glomerulonephritis type II, MPA Microscopic polyangiitis, NA not applicable/ not assessed, IFTA interstitial fibrosis/tubular atrophy, GS glomerular sclerosis, II interstitial inflammation. Median, Mean and standard deviation (SD) for the patients with follow-up and from stable (S) and progressive (P) patients are shown at the bottom of the table. Age groups have been defined as follows: group 1 = age <30 years, 2 = 30–45, 3 = 46–60, 4 = age >60 years. p-values indicate statistical significance comparing clinical data (age, follow-up, creatinine, proteinuria) from stable and progressive patients.

**Table 2:** **Patient characteristics – validation cohort**

**Patient**	**Age group**	**Follow-up time [months]**	**Creatinine at time of biopsy [mg/dl]**	**Creatinine at follow-up [mg/dl]**	**eGFR (MDRD) at time of biopsy [ml/min/1.73 m** ^**2**^ **]**	**eGFR (MDRD) at follow-up [ml/min/1.73 m** ^**2**^ **]**	**Proteinuria at time of biopsy [g/d]**	**Proteinuria at follow-up [g/d]**	**Histological diagnosis**	**SD/PD**	**Endpoint**	**IFTA**	**GS**	**II**
1	2	5	4.60	6.67	<20	<20	4.8	2.0	IGAN	Progressive	ESRD	Moderate	4	Moderate
2	2	51	0.88	0.96	>60	>60	1.9	1.5	IGAN	Stable		Moderate	2	Moderate
3	3	34	1.34	1.97	41.4	26.2	2.2	0.5	LN IV	Stable		Moderate	3	Moderate
4	1	23	1.09	0.98	>60	>60	3.0	0.3	LN IV	Stable		Moderate	2	Moderate
5	1	50	0.52	0.86	>60	>60	10.4	0.2	LN IV	Stable		Moderate	0	Moderate
6	1	41	1.05	5.46	>60	<20	1.7	3.3	IGAN	Progressive	ESRD	Moderate	0	None
7	3	58	0.86	8.54	>60	<20	1.3	4.6	MGN	Progressive	ESRD	Moderate	0	None
8	4	31	1.13	2.84	>60	22	2.5	2.8	MGN	Progressive	2xCrea	Moderate	0	None
9	3	25	2.59	6.61	25.5	<20	NA	NA	FSGS	Progressive	ESRD	Severe	4	NA
10	3	72	1.43	7.60	53.2	<20	NA	5.2	IGAN	Progressive	ESRD	Moderate	0	None
11	3	40	1.10	0.78	52.4	>60	NA	0.0	IGAN	Stable		Moderate	3	Moderate
12	3	1	4.86	6.34	<20	<20	7.9	12.5	IGAN	Progressive	ESRD	Severe	4	None
13	3	23	1.62	1.59	44.3	45.1	4.8	0.8	IGAN	Stable		Severe	1	Moderate
14	2	7	0.91	0.82	>60	>60	10.0	0.8	IGAN	Stable		None	0	None
15	4	17	1.23	4.32	58.3	<20	6.3	6.5	IGAN	Progressive	2xCrea	Moderate	2	None
16	3	16	2.71	4.16	24.2	<20	1.0	1.1	IGAN	Stable		Severe	3	Severe
Median	50	28	1.18	3.50			3.0	1.5						
Mean	47	31	1.75	3.78			4.4	2.8						
SD	15	20	1.30	2.76			3.3	3.3						
Median S	46.5	28.4	1.1	0.97			3.0	0.6						
Median P	49.5	28	1.33	6.48			3.6	4.6						
Mean S	43.9	30.4	1.27	1.52			4.8	0.7						
Mean P	49.8	31.4	2.22	6.05			4.1	5.3						
SD S	12.9	16	0.67	1.15			3.9	0.5						
SD P	16.9	24.8	1.64	1.81			2.7	3.5						
p-value	n.s.	n.s.	n.s.	<0.001			n.s.	0.01						

Abbreviations see legend of Table 1.

### General statistics

The following procedure was carried out to test significance of findings: Values from stable and progressive patients were tested for Gaussian distribution using Kolmogorov-Smirnov- Test unless categorical data. In case of non-Gaussian distribution non-parametric unpaired Kruskal-Wallis test was applied. In all other cases Student’s T-Test was used. P values below 5% were defined as statistically significant.

## Results

### Establishment of a biomarker model predictive for follow-up creatinine – discovery cohort

#### Patient characteristics

Detailed patient characteristics were published previously and are summarized in Table 1 [8,14]. The proportion of female patients was 37%. Ten patient samples showed a CREDI of less than or equal to 2, seven samples had a CREDI of 3 to 4, and ten samples showed severe damage with a CREDI score above 4. Twenty-three samples had INSCO values of 0 or 1, three samples had a score of 2, and one patient sample was scored with a value of 3. There was a significant correlation between CREDI and INSCO (R = 0.57, p < 0.05). Twenty patients with sufficient clinical follow-up data were included in the subsequent correlation analysis of CREDI and INSCO associated genes with kidney function during follow-up. There were no significant differences in serum creatinine and proteinuria at time of biopsy, and neither in the CREDI nor in the INSCO values between subjects with and without follow-up data. The same non-significant results were obtained comparing stable versus progressive patients (including follow-up time), despite a significant difference in the creatinine at follow-up and INSCO values (Median INSCO(progressive) = 2, INSCO(stable) = 0, p = 0.004).

#### Correlation of gene expression with chronic renal damage index

Transcriptomics profiles from the 20 samples were correlated to CREDI. The expression status of 562 unique genes correlated significantly with CREDI. Of these 562 genes 222 showed a positive correlation (i.e. being upregulated) with increasing damage score, and 340 genes showed a negative correlation (i.e. being downregulated) with increasing damage score, respectively (Additional file 1: Table S1). Pathways found to be enriched either in positively or negatively correlated genes are summarized in Table 3. Among others the *insulin-like growth factor*-, the *transforming growth factor-,* and the *integrin signaling-*pathway were significantly enriched with genes in the list of 222 positively correlated genes. Using the 340 downregulated genes we identified 27 significantly enriched pathways. These pathways included, among others, metabolism (e.g. *ATP synthesis*) signaling (e.g. *FGF-signaling*, *EGF receptor signaling, VEGF-signaling, angiogenesis*), and inflammation (e.g. *T-cell activation, B-cell activation*).

**Table 3:** **Pathways enriched/depleted in differentially regulated genes correlating with CREDI and INSCO**

**Pathways enriched/depleted in differentially regulated genes correlating with CREDI**		
**Upregulated genes (n = 222)**		
**Pathways**	**# Genes**	**P value**
Hedgehog signaling pathway	3	0.002
Insulin/IGF pathway-mitogen activated protein kinase kinase/MAP kinase cascade	3	0.006
Formyltetrahydroformate biosynthesis	2	0.006
PDGF signaling pathway	6	0.006
TGF-beta signaling pathway	5	0.018
Transcription regulation by bZIP transcription factor	3	0.018
Muscarinic acetylcholine receptor 2 and 4 signaling pathway	3	0.027
Ornithine degradation	1	0.030
Integrin signaling pathway	5	0.040
Serine glycine biosynthesis	1	0.050
**Downregulated genes (n = 340)**		
**Pathways**	**# genes**	**P value**
ATP synthesis	4	0.000
FGF signaling pathway	8	0.001
EGF receptor signaling pathway	8	0.002
GABA-B_receptor_II_signaling	4	0.004
Alzheimer disease-amyloid secretase pathway	5	0.006
T cell activation	6	0.006
Endogenous_cannabinoid_signaling	3	0.007
Histamine H1 receptor mediated signaling pathway	4	0.007
Metabotropic glutamate receptor group II pathway	4	0.009
B cell activation	5	0.010
Axon guidance mediated by netrin	3	0.012
Endothelin signaling pathway	5	0.015
Oxytocin receptor mediated signaling pathway	4	0.016
Thyrotropin-releasing hormone receptor signaling pathway	4	0.018
Muscarinic acetylcholine receptor 2 and 4 signaling pathway	4	0.018
Cholesterol biosynthesis	2	0.018
Metabotropic glutamate receptor group I pathway	3	0.020
5HT2 type receptor mediated signaling pathway	4	0.025
Succinate to proprionate conversion	1	0.031
VEGF signaling pathway	4	0.032
5HT1 type receptor mediated signaling pathway	3	0.033
Angiogenesis	7	0.033
Inflammation mediated by chemokine and cytokine signaling pathway	9	0.037
PDGF signaling pathway	6	0.042
DNA replication	2	0.044
Cell cycle	2	0.048
Heterotrimeric G-protein signaling pathway-Gi alpha and Gs alpha mediated pathway	6	0.050
**Pathways enriched/depleted in differentially regulated genes correlating with INSCO**		
**Upregulated genes (n = 150)**		
**Pathways**	**# Genes**	**P value**
Chorismate biosynthesis	1	0.000
Nicotinic acetylcholine receptor signaling pathway	3	0.033
Mannose metabolism	1	0.049
**Downregulated genes (n = 135)**		
**Pathways**	**# Genes**	**P value**
mRNA splicing	2	0.002
General transcription by RNA polymerase I	2	0.007
PLP biosynthesis	1	0.013
Leucine biosynthesis	1	0.013
Alanine biosynthesis	1	0.013
Vitamin B6 metabolism	1	0.019
Valine biosynthesis	1	0.019
Isoleucine biosynthesis	1	0.019
PDGF signaling pathway	4	0.019
Serine glycine biosynthesis	1	0.031
Histamine H1 receptor mediated signaling pathway	2	0.037
p53 pathway feedback loops 2	2	0.044
Apoptosis signaling pathway	3	0.045

Biological pathways being enriched or depleted with respect to the set of significantly correlated genes were analyzed using the PANTHER (**P**rotein **AN**alysis **TH**rough **E**volutionary **R**elationships) Classification System. # genes … number of genes.

#### Correlation of gene expression with inflammation score

Next, we correlated gene expression data from the same 20 samples to INSCO. The expression values of 285 genes correlated with INSCO, of which 150 showed a positive and 135 showed a negative correlation (Additional file 1: Table S2). In the 150 genes three pathways were significantly overrepresented, namely the *chorismate biosynthesis*-, the *nicotinic acetylcholine receptor signaling*-, and the *mannose metabolism*-pathway. Thirteen pathways were identified on the basis of the 135 negatively correlated genes (Table 3). Several pathways linked to amino-acid synthesis such as *leucine*-, *alanine*-, *valine-*, *isoleucine-* and *serine glycine biosynthesis*-pathways. Furthermore, *mRNA splicing*- and *general transcription by RNA polymerase I*-pathways were identified as being enriched with down regulated genes.

#### 2-step approach for identification of candidate genes for prediction of renal outcome

We correlated the expression values of genes showing statistically significant association to CREDI (562 genes) and INSCO (285 genes) with follow-up creatinine. Significant correlation (p < 0.05) was identified for 39 genes from the CREDI group, and for 90 genes from INSCO set of genes, respectively. To maximize the predictive value of selected genes we applied a 2-step approach: Kidney specificity of the genes was evaluated using information as provided by the SOURCE tool (http://source.stanford.edu). Five (CREDI) and ten (INSCO) genes were found to be highly expressed in human kidney (Table 4). We further evaluated the pair-wise correlation of expression to identify those candidate genes showing the lowest correlation in expression, thus having an independent predictive value for follow-up creatinine. Four CREDI genes (vascular endothelial growth factor C (VEGF-C), podoplanin (PDPN), semaphorin 6A (SEMA6A), integrin beta 6 (ITGB6)), and six INSCO genes (thrombospondin 1 (THBS1), tribbles homolog 1 (TRIB1), bone morphogenetic protein 7 (BMP7), chordin-like 1 (CHRDL1), apoptosis-inducing factor 1 (AIFM), syntaxin 7 (STX7)) were identified following this approach.

**Table 4:** **Top candidate marker genes for prediction of creatinine at follow up**

**Symbol**	**Genelist**	**Corr. FU-Crea.**	**p-value**
THBS1	INSCO	0.781	0.000
TRIB1	INSCO	0.776	0.000
NCF1C	INSCO	0.675	0.001
SEMA6A	CREDI	0.522	0.038
PDPN	CREDI	0.495	0.026
SPP2	CREDI	0.485	0.048
ITGB6	CREDI	0.471	0.036
GREM1	INSCO	−0.447	0.048
BMP7	INSCO	−0.465	0.039
AIFM1	INSCO	−0.572	0.013
CTH	INSCO	−0.578	0.015
PRPH	INSCO	−0.603	0.010
STX7	INSCO	−0.631	0.004
VEGFC	CREDI	−0.636	0.003
CHRDL1	INSCO	−0.665	0.002

These genes represent the top candidates which correlated with histological damage (CREDI or INSCO) and creatinine at follow up, and which are known to be expressed in kidney tissue.

#### Linear regression analysis

The predictive value of classical markers, creatinine at time of biopsy and histopathological grading, was calculated, and the additive predictive value of a gene-panel of CREDI or INSCO genes was calculated. Adjusted R^2^ values of the classical markers, creatinine at time of biopsy, CREDI and INSCO were 0.18, 0.02 and 0.44 with p-values of 0.0391, 0.2699 and 0.0012, respectively. Hence, only INSCO but not CREDI classification was able to predict 44% of variation of follow-up creatinine with statistical significance. The best model based solely on traditional markers consisted of creatinine at time of biopsy and INSCO with an adjusted R^2^ of 0.51 (p = 0.0014) (Table 5). The best model after a step-wise selection using all traditional markers and the CREDI genes resulted in a model consisting of VEGF-C and INSCO with a comparable predictive value of 0.51 (p = 0.0009). Using the INSCO genes together with the traditional parameters resulted in a model consisting of the three genes thrombospondin 1 (THBS1), bone morphogenetic protein 7 (BMP7), and tribbles homolog 1 (TRIB1). The predictive value of this model was 0.94 (p < 0.0001), thus predicting 94% of the variation of follow-up creatinine for the given sample set with a median follow-up time of 49 months (Table 5). The bias corrected bootstrap confidence interval for the coefficient of determination was 0.558 – 0.987.

**Table 5:** **Linear regression analysis**

	**Creatinine at follow-up**		
**Best single clinical parameter**	**Parameter estimate**	**p-value**	**adj R** ^**2**^
**INSCO**	1.799	0.0012	
**Total**		**0.0012**	**0.44**
	**Creatinine at follow-up**		
**Best clinical parameter combination**	**Parameter estimate**	**p-value**	**adj R** ^**2**^
**INSCO**	1.472	0.0065	
**Crea_biopsy**	0.755	0.0834	
**Total**		**0.0014**	**0.51**
	**Creatinine at follow-up**		
**Clinical parameters + CREDI genes**	**Parameter estimate**	**p-value**	**adj R** ^**2**^
**INSCO**	1.277	0.0248	
**VEGFC**	−0.812	0.0268	
**Total**		**0.0009**	**0.51**
	**Creatinine at follow-up**		
**Clinical parameters + INSCO genes**	**Parameter estimate**	**p-value**	**adj R** ^**2**^
**BMP7**	−0.67	0.0015	
**THBS1**	1.160	0.0035	
**TRIB1**	0.534	0.0007	
**Total**		**< 0.0001**	**0.94**

Adjusted R2- and p-values for different models for prediction of follow-up creatinine are shown.

These three genes were found to be significantly differentially expressed when comparing array data from stable and progressive patients. In progressive patients we found a significantly higher expression of THBS1 (p = 0.009) and TRIB1 (p = 0.011), whereas the expression levels of BMP7 were significantly lower (p = 0.007) as compared to stable patients.

Using hard renal endpoints (see Table 1), e.g. doubling of serum creatinine or ESRD, for definition of progressive kidney disease we have calculated sensitivity and specificity for each marker using ROC analysis. Sensitivity/specificity was 1.00/1.00 for THBS1 (cut-off: 1.17), 0.67/0.83 for TRIB1 (cut-off: 2.42) and 0.93/0.68 for BMP7 (cut-off: −1.15), respectively.

### Validation cohort

#### Patient characteristics

In order to validate the established biomarker model microarrays were performed using whole kidney biopsies of a 2^nd^ cohort of patients with proteinuric kidney diseases (n = 16). The patient characteristics are shown in detail in Table 2. This cohort consisted of 25% females. There was no significant difference in age, follow-up time, creatinine/proteinuria at time of biopsy comparing stable and progressive patients. Comparing the clinical data with those from the discovery cohort we found also no significant difference, except a shorter follow-up time (49 vs. 28 months).

#### Linear regression analysis

The predictive value of the model creatinine at time of biopsy plus the three biomarkers of the INSCO-panel was calculated. The predictive value of this model was 0.4852 (p = 0.0115), thus predicting 48% of the variation of follow-up creatinine for the given sample set with a median follow-up time of 28 months. Comparing the predictive value of the traditional parameter creatinine at time of biopsy with the 3-biomarker panel yielded in a significantly better prediction of follow-up creatinine using the 3 biomarkers (p = 0.0236).

Again, these three genes were found to be differentially expressed when comparing array data from stable and progressive patients; however only TRIB1 reached statistical significance (p = 0.023).

We have calculated sensitivity and specificity for each marker using ROC analysis in this cohort. Sensitivity/specificity was 1.000/0.625 for THBS1 (cut-off: 10.09), 0.625/0.750 for TRIB1 (cut-off: 8.03) and 0.875/0.625 for BMP7 (cut-off: 6225), respectively.

## Discussion

In this project we performed a transcriptomics approach to identify tubular gene expression profiles associated with (i) glomerulosclerosis, tubular atrophy or interstitial fibrosis, or (ii) interstitial inflammatory infiltration. The histopathological features of tubular atrophy and interstitital fibrosis correlate better with poor renal prognosis compared to glomerular lesions, which is accordance with previously published data. The identified activation of features assigned to *IGF-*, *PDGF-*, *TGF beta*- and *integrin*-pathways in fibrotic tissue is in line with data from various groups [21-23]. More interestingly, a number of downregulated features was enriched in specific pathways including several *receptor-* and *intracellular signaling-*, *metabolism-*, *cell cycle-* and *angiogenesis-*pathways. The impact on *ATP-synthesis-* and *cell cycle-*pathways is in accordance with data showing that hypoxia in particular in proximal tubule cells depletes the cells of ATP, induces mitochondrial fragmentation, and finally leads to apoptosis [24].

We next correlated tubular gene expression with the presence of interstitial inflammation. Several pathways linked to *amino acid synthesis*, *mRNA-splicing*, *transcription*, *PDGF-signaling*, *apoptosis* and *p53-signaling* showed enrichment in downregulated genes. Seven of the 13 pathways were defined as significantly overrepresented because of the presence of two genes: phosphoserine aminotransferase 1 (PSAT1) and branched chain amino-acid transaminase 2, mitochondrial (BCAT2) (data not shown). PSAT1 as a member of the *PLP biosynthesis* and the *serine glycine biosynthesis* pathway is involved in serine, glycine and threonine metabolic pathways, and also in the biosynthesis of vitamin B6 (pyridoxine). BCAT2 is expressed in mitochondria and catalyzes the first step in the production of the branched amino-acids leucine, isoleucine and valine. These results may emphasize the role of mitochondrial dysregulation in CKD [24-26].

In the next step we aimed at the identification of novel molecular markers indicative for progression of chronic renal failure. Since the extent of tubulointerstitial fibrosis represents an established risk factor for kidney disease progression, we hypothesized that genes from the CREDI panel will outperform genes from the INSCO panel regarding the predictive value. Surprisingly, only 39 of 562 (7%) CREDI genes also correlated with serum creatinine during follow up, while 90 of 285 (32%) INSCO genes showed a significant correlation. Furthermore, INSCO itself predicted 44% of follow-up creatinine variation (p = 0.0012), while the predictive value of CREDI was not significant. It has been generally accepted that tissue fibrosis and inflammation both contribute to progressive renal scarring and are finally associated with renal function decline [27]. Although some uncertainty exists about details of the causal and chronological relationship, it has been generally proposed that renal injury is followed by recruitment of inflammatory cells, release of fibrogenic cytokines, and finally the activation of collagen-producing cells [28]. Our results of significant correlation of inflammation but not fibrosis with renal function decline point towards a biopsy bias favouring acute inflammatory glomerulonephritis rather than slowly progressing fibrosing renal disease. On the other hand these findings might also be compatible with the chronological sequence of inflammation followed by fibrosis processes.

We identified a panel of three genes which were highly predictive for the variation of follow-up creatinine after a median follow up time of more than 4 years. Using the expression values of THBS1, BMP7 and TRIB1 we were able to predict an additional 43% of creatinine variance on top of traditional progression markers, being creatinine and inflammation score at time of biopsy. Again, all three genes were identified from the inflammation gene panel further emphasizing the relevance of tissue inflammation in progressive renal failure.

In order to validate this model found in microdissected renal proximal tubule cells, we performed microarrays from whole kidney biopsies in a 2^nd^ cohort of patients suffering from proteinuric kidney diseases. We decided to use whole kidney biopsies by purpose, as LCM of renal proximal tubule cells is not a standard procedure in histopathological work-up after kidney biopsy and we were interested if genomic patterns derived from LCM proximal tubule cells can be found in the background signal from all other cell types in whole kidney biopsies. Using our 3-biomarker model we were able to predict 48% of creatinine variance at follow up (p = 0.0115).

We used creatinine at follow-up as the clinical outcome parameter in this project, since worsening of creatinine (e.g. doubling of serum creatinine) and reaching end-stage renal disease (ESRD) are the only established endpoints in clinical renal research approved by the food and drug administration (FDA). Alternatively, one can use eGFR or eGFR slope as parameter for changes in kidney function, but eGFR formulas (MDRD, CKD-EPI) are not reliable at eGFR values above 60 and below 20 ml/min/1.73 m^2^ [29-31]. In our cohorts (Tables 1 and 2) a substantial number of patients are in these GFR ranges, hence we did not correlate gene expression data with eGFR or eGFR slope. However, creatinine at follow-up showed a strong and significant correlation (Pearson R = 0.848, p < 0.0001) with creatinine slope, and also the three identified biomarker candidates showed a significant correlation with creatinine slope (Pearson R = 0.722 (THBS1, p = 0.038), 0.685 (TRIB1, p = 0.026), −0.562 (BMP7, p = 0.010)). These results further underline the validity of these three marker candidates.

The primary approach of this study was to establish a model to predict serum creatinine levels at the follow-up time point. By establishing multiple linear regression models using continuously distributed biomarker candidates and gold standard parameters (e.g. creatinine or proteinuria at time of biopsy), we identified a model consisting of 3 biomarkers (THBS1, TRIB1 and BMP7). In addition we calculated sensitivity and specificity of each of these markers to facilitate the interpretation of our data in the context of published literature [32,33]. For this purpose we defined progression of chronic kidney disease using established parameters, i.e. doubling of serum creatinine and ESRD. Furthermore, best cut-off values for the single markers were calculated using Youden’s statistics [20]. We found that THBS1 was the best marker, followed by BMP7 and TRIB1. It was surprising that THBS1, providing a sensitivity and specificity of 1.00 in the discovery cohort, performed that well. However, given the limited number of patients investigated these results should not be generalized to other populations without caution. We are currently planning to establish a staining of our three biomarkers in patient kidney biopsy samples in order to prospectively address the predictive value of this biomarker.

Concerning the role of TRIB1, Kiss-Toth and co-workers first described tribbles homologes as MAPK activity controlling proteins [34]. Recently, it was shown that TRIB1 might be utilized in renal allografts as biomarker for chronic antibody-mediated rejection [35]. Furthermore, TRIB1 expression was correlated to non-diabetic end-stage renal disease [36]. BMP7 is a member of the TGF-beta superfamily and has been shown to be implicated in regulation of renal function and determination of the number of renal progenitor cells [37]. In particular, various groups have described the pivotal role of BMP7 in EMT, but results are controversial. Xu et al. [38,39] published data providing evidence that BMP7 exerts antifibrotic effects via blocking and reversing TGF-beta 1 induced EMT, whereas Dudas and colleagues did not find an inhibitory effect of BMP7 on TGF-beta 1 mediated EMT [40]. In a previous project we were able to show increased expression of BMP7 mRNA and protein in renal tubule cells in biopsies from proteinuric renal diseases as compared to controls [14]. However, most of the patients included in this former study showed a stable course of kidney disease with virtually no decline of kidney function over time. Recently, a low BMP7 RNA levels were proposed to be an early marker of renal allograft dysfunction [41]. In amyloidosis patients Denizli et al. described a non-significant correlation of high levels of BMP7 and CKD progression [42].

Thrombospondin 1 is a subunit of a disulfide-linked homotrimeric protein. This protein is an adhesive glycoprotein that mediates cell-to-cell and cell-to-matrix interactions. Hugo et al. [43] have shown that THBS1 precedes and is an early marker for the development of tubulointerstitial kidney disease, which might be explained by its role as an endogenous activator of TGF-beta in type 1 diabetes [44] and fibrotic renal disease [45]. Cui et al. have shown that THBS1 is an important mediator of obesity-induced kidney dysfunction [46]. Recently, a THBS1 short hairpin RNA suppressed peritubular capillary injury and tubulointerstitial fibrosis in unilateral ureteral obstruction (UUO)-induced renal fibrosis [47].

To summarize, THBS1 and BMP7 are involved in TGF beta pathways, which have been shown to be activated in progressive CKD.

This paper has several limitations. More clinical and demographic data are needed to adjust the calculated models to confounders, such as hypertension or hypercholesterolemia. However, it was not possible to assess this information from the patients’ records. Another interesting issue would have been to analyse the gene expression patterns in response to therapeutic interventions. First, the cohorts and the interventions (if there were any at all) were to heterogenous to collect and to analyze data in a reliable manner. Second, some of the patients were not treated at our center, so a significant proportion of intermediate follow-up data is missing. And third, we did not continuously perform kidney biospies during the follow-up, so the outcome of the interventions on the histological and genomic level is unclear. Hence, it was not possible to investigate the predictive value of changes of gene expression in response to treatment.

We correlated gene expression data with the variability of creatinine at follow-up, which might be a very hard endpoint and thus more subtle transcriptomic changes are missed. Due to the limitations of eGFR mentioned above we did not correlate the expression of the transcripts with delta eGFR. However, the significant correlation between creatinine at follow-up and creatinine slope, and between the expression values of the three biomarker candidates and creatinine slope corroborates our results.

Certainly, the degree to which the 3-gene panel can be generalised to other chronic kidney disease cohorts needs to be tested in further studies in larger validation sample sets, as the predictive power delineated on the given cohort is probably an overestimation of the true predictive value due to the small size of the 1^st^ study cohort. However, the bootstrap sampling using 2000 case resampled datasets resulted in a bias corrected confidence interval of 0.558 – 0.987, suggesting nevertheless a higher predictive value of these 3 genes than a combination of traditional parameters such as creatinine and INSCO. The procedure for delineating such gene sets as presented in this work might well be applicable for larger cohorts. Additionally, we have analysed the 3-biomarker model in a 2^nd^ validation cohort of patients and also found a predictive value, which was statistically significant, for follow-up creatinine.

## Conclusion

We identified distinct gene expression profiles from laser-capture microdissected renal tubule cells associated with chronic renal damage or inflammation. The 3-gene panel THBS1, BMP7 and TRIB1 from the inflammation gene panel predicted follow-up creatinine significantly better than traditional markers such as serum creatinine at time of biopsy and the presence of inflammatory infiltrates in the biopsy. These data were validated using gene expression profiles from whole kidney biopsies in a second cohort of patients.

## Additional file

Additional file 1: Tables S1 and S2. Show the 562 and 285 genes that were either negatively or positively correlated with CREDI and INSCO, respectively. Gene names, accession numbers, UniProt ID, Gene IDs, GO-Terms (abbreviated) and a few other possible important information concerning the respective genes are listed in the first 12 collumns. Diagnosis and CREDI/INSCO – values are listed in the first 2 lines above each patient (patient numbers (as in Table 1) are listed in the third line above expression values). The last 5 collumns show the number of missing expression values per transcript (blanks) and if at least 80% of signals in the microarray per gene met our quality standards (80% filter; 1=yes, 0=no); furthermore correlation coefficient (Pearson) and t- and p-values are listed for each gene.
